# Diagnostic Utility of the “Air Sign” as a Radiological Indicator for Mandibular Body and Angle Fractures

**DOI:** 10.3390/jcm13206288

**Published:** 2024-10-21

**Authors:** Weronika Michalik, Joanna Kuczera, Jakub Bargiel, Krzysztof Gąsiorowski, Tomasz Marecik, Paweł Szczurowski, Grażyna Wyszyńska-Pawelec, Michał Gontarz

**Affiliations:** 1 Students’ Scientific Group of the Department of Cranio-Maxillofacial Surgery, Jagiellonian University Medical College, 30-688 Cracow, Poland; weronika.michalik@student.uj.edu.pl (W.M.); joanna.kuczera@student.uj.edu.pl (J.K.); 2 Department of Cranio-Maxillofacial Surgery, Jagiellonian University Medical College, 30-688 Cracow, Poland; jakub.bargiel@uj.edu.pl (J.B.); krzysztof.gasiorowski@uj.edu.pl (K.G.); tomasz.marecik@uj.edu.pl (T.M.); pawel.szczurowski@uj.edu.pl (P.S.); grazyna.wyszynska-pawelec@uj.edu.pl (G.W.-P.)

**Keywords:** mandibular fractures, cone beam computed tomography, computed tomography, preoperative diagnostics, facial trauma, radiological examination

## Abstract

**Background:** To plan optimal treatment, obtain satisfactory outcomes, and avoid undesirable clinical errors, surgeons need to have efficient tools for providing a complete and prompt diagnosis. The aim of this study was to establish the sensitivity, specificity, positive and negative predictive values, false positive rate, and false negative rate of the “air sign” (AS) within soft tissues as an indirect radiological indicator of mandibular body and angle fractures. **Methods:** A retrospective analysis of preoperative computed tomography (CT) and cone beam computed tomography (CBCT) scans was performed on patients with mandibular fractures within a three-year period. Two fracture types were analyzed: open and closed fractures. **Results:** Forty-three patients with a total of 71 mandibular fractures were included in the study. The mean age of the patients was 35 years, and the majority were male (83.7%). The sensitivity of the AS was 92.2%, specificity = 90.0%, positive predictive value = 95.9%, negative predictive value = 81.8%, false positive rate = 10.0%, and false negative rate = 7.8%. Higher values were observed for open fractures compared to closed fractures. **Conclusions:** The sensitivity and specificity of AS are lower than those of OPG, CT, and CBCT. However, AS offers an important additional radiological indicator that can effectively reduce the risk of misdiagnosing mandibular body and angle fractures.

## 1. Introduction

The mandible is the most common site of injury within the facial skeleton, accounting for 40% to 62% of facial fractures. Over half of these cases are diagnosed as multiple fracture lines, with double fracture lines being the most prevalent [1]. While mandibular fractures are relatively common, their prevalence and injury mechanisms vary depending on cultural, socioeconomic, and demographic factors. The demographic most frequently affected is the male population in their third decade of life, who are often distressed as a result of interpersonal violence, frequently associated with inebriation [2]. In contrast, in the female population, mandible trauma is more often the result of road accidents or falls [2,3]. Nevertheless, it is worth expanding the analysis to probable domestic violence or suicidal history, especially if there is any evidence of intentional injuries.

In the past, conventional plain radiography for concomitant injuries was focused primarily on bone tissue, without the ability to provide detailed imaging of soft tissue injuries. This technique enabled the detection of bone fractures with 86% sensitivity at a relatively low cost, making it a popular choice for diagnosing trauma patients [4]. However, conventional radiographic evaluation has some limitations, including the number of projections that must be taken for a complete analysis of a fracture line. The uncontrolled movements of an unstable patient’s neck and head could potentially result in the generation of radiological artifacts during the diagnostic process. The relatively high occurrence of artifacts, superimposed images, and detail obscurity, particularly in cases of condylar and symphyseal fractures, make conventional radiography a less favorable option in trauma centers [5]. Consequently, computed tomography (CT) and cone beam computed tomography (CBCT) have emerged as invaluable tools in this context. During a single scanning process, highly accurate and high-resolution images can be obtained in all anatomical planes. Additionally, three-dimensional (3D) reconstructions can be created, which provide a broader perspective and facilitate the optimization and individualization of medical treatment. Recent research indicates that artificial intelligence (AI) tools, particularly deep learning systems, are being integrated into clinical practice with the objective of enhancing the diagnostic efficacy of CT and CBCT modalities [6].

Furthermore, CT imaging is an effective method for visualizing soft tissue abnormalities, including foreign bodies, hematomas, muscles damage, nerves damage, and the presence of air bubbles. The presence of air bubbles in soft tissue may indicate the existence of skin or mucosal wounds, organic foreign bodies, and, indirectly, the possibility of facial skeleton fractures. Particularly, a case of air bubbles in the orbit may indicate orbital wall fractures [7]. Also, air accumulation in the upper and middle face area may occur in fractures of the frontal sinus or maxillary sinus walls, often noted in zygomaticomaxillary complex or LeFort fractures. Additionally, the air around the styloid process of the temporal bone could indicate a temporal bone fracture, and pneumocephalus may be present in skull base fractures [8]. 

In a previous study, a review of medical charts from a one-year period revealed that 4% of mandibular body fractures were overlooked by radiologists and surgeons prior to surgery [9]. The presence of air bubbles within soft tissues, identified through CT/CBCT imaging, indicated the presence of an underlying, misdiagnosed fracture without dislocation. This phenomenon has been termed as “air sign” (AS) [9]. In the case of mandibular body/angle fractures, the stretching and crushing forces cause bone deformation, and, eventually, a bone fracture may also result in the disruption of the continuity of the mucous membrane, gingiva, or dental sockets. In addition to the previously mentioned factors, the presence of negative pressure within the surrounding soft tissues may result in the aspiration of the air into these areas. Furthermore, it is important to note that air bubbles visible in soft tissues at CT or CBCT images may originate from skin wounds, significant subcutaneous emphysemas, or the patient’s saliva [9]. Therefore, such false positive results of the AS suggest that a full clinical examination is essential for the accurate diagnosis of a potential mandibular body or angle fracture. As this phenomenon has not been described in mandibular fracture in any previous works so far, there is no evidence to comparatively support our finding. We decided to describe its radiological significance, clinical utility, and possible interoperation with the existing diagnostic methods.

The objective of the study is to evaluate the sensitivity, specificity, positive predictive value, and negative predictive value of the AS as a radiological indicator for mandibular body and angle fractures in CT and CBCT evaluations.

## 2. Materials and Methods

### 2.1. Characteristics of the Study Group

**Figure 1:**
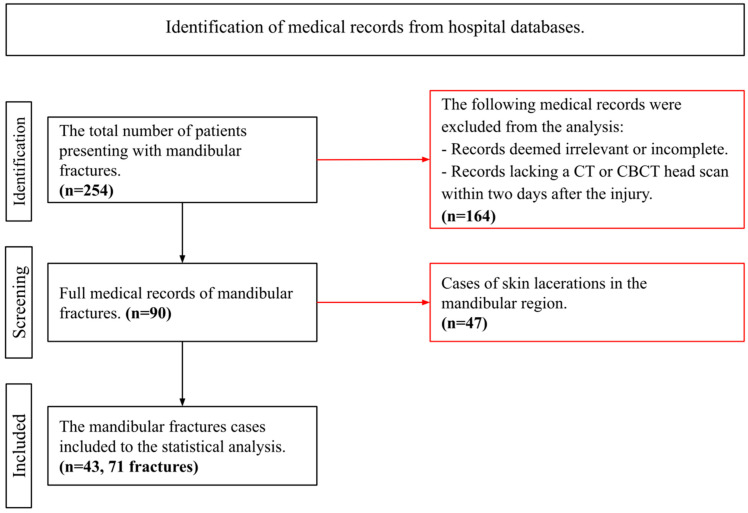
Flow chart of the process of establishing the study group. Identification of medical records from hospital databases.

To determine the sensitivity, specificity, positive predictive value (PPV), and negative predictive value (NPV) of this diagnostic indicator, a retrospective analysis of 254 medical records of mandibular fractures at the Department of Cranio-Maxillofacial Surgery, University Hospital in Cracow, Poland, was conducted over the period from January 2021 to July 2024. The presence of the AS was evaluated based on preoperative CT or CBCT scans of the viscerocranium. Only patients with CT or CBCT scans performed within 2 days postinjury were considered eligible for analysis. The process of identifying the study group was presented in a graphical format (Figure 1).

Inclusion criteria were:

(1) Isolated mandibular fractures or resulting from panfacial fractures;
(2) CT/CBCT performed no later than 2 days after the injury;

No lacerations or other wounds of the facial skin in the mandibular body area.

Exclusion criteria were:

(1) Isolated fractures of the upper and middle facial unit;
(2) CT/CBCT performed more than 2 days after the injury;
(3) Incomplete medical data available in hospital’s electronic database.

### 2.2. Study Design

Two types of fractures included for statistical analysis were established:

1. Open fractures: located within the body/angle of mandible;
2. Closed fractures: located within the ramus, condylar/coronoid process of mandible.

When a patient had multiple fracture lines within one structure of the mandible on the same side, it was considered as one result. Each affected side (left and right) was evaluated separately; thus, the number of results exceeded the number of included patients. 

The evaluation of the AS for each fracture type was conducted in two established, nonmixable configurations:

1. Body of the mandible + unilateral/bilateral condylar/coronoid process or ramus of the mandible;
2. Left/right body/angle of the mandible + contralateral body/angle of the mandible.

In those cases where a diagnosed fracture of the mandibular body or angle was present, the air bubbles were evaluated within the soft tissues in the area of the fracture site and in the region of the contralateral mandibular body and angle. In the case of a mandibular ramus, coronoid, condylar process fracture, the presence of air bubbles within the soft tissues in proximity to the mandibular body and angle was evaluated. 

### 2.3. Statistical Classification

The radiological evaluation was conducted cooperatively by three respective researchers (W.M., J.K., M.G.). The results were divided into four categories based on specific criteria to enable statistical analysis of the air sign.

1. True positive (TP)—diagnosed body/angle fracture + presence of AS in soft tissues.

**Figure 2:**
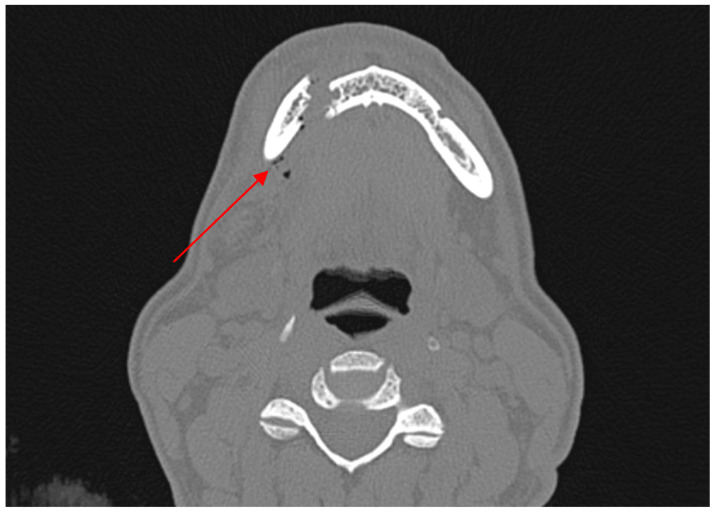
A 49-year-old male patient presented with a fracture of the mandible body on the right and the angle of the mandible on the left. The presence of air bubbles can be observed in the soft tissues correlating with the area of the fracture (red arrow).

Cases in which the air within the soft tissues was noted near the mandibular body or angle in the configurations described in the study design (Figure 2).

2. False positive (FP)—no body/angle fracture + presence of AS in soft tissues.

**Figure 3:**
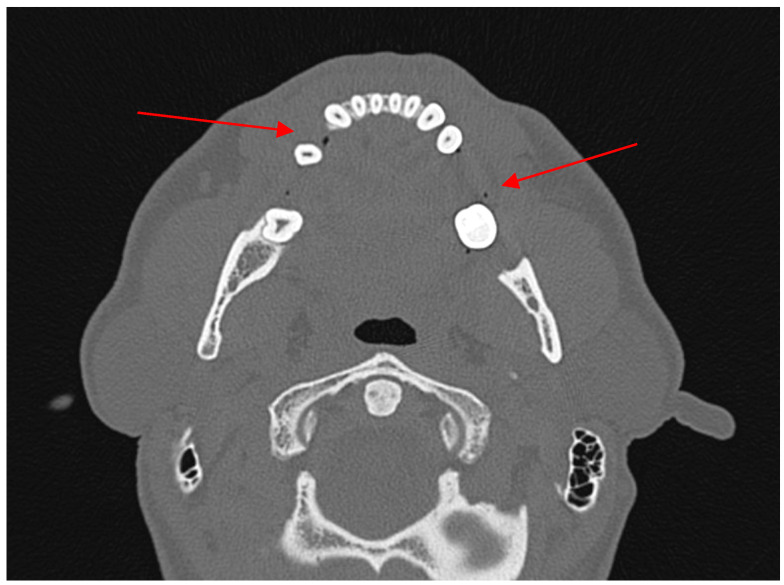
A 47-year-old patient diagnosed with a fracture of the right condylar process of the mandible. The CT scan reveals the presence of air bubbles at the level of the necks and crowns of the teeth (red arrows). Given their position, it can be determined that these are air bubbles associated with saliva in the oral cavity.

A false positive result indicates the presence of an air bubble in the absence of a fracture. The air bubble may originate from saliva situated beneath the gingival margin of the teeth, within the vestibule of the oral cavity, or on the floor of the mouth (Figure 3). Patients exhibiting an air bubble in the vestibule of the oral cavity at the level of the necks or crowns of the teeth were excluded from the study, as it was evident, based on anatomical considerations, that the air bubble was not situated within the soft tissues surrounding the mandible. Another false positive is evident in panfacial fractures that involve the upper and middle facial unit, where air bubbles in soft tissue near to the mandible may manifest as a result of fractures of the paranasal sinuses. Additionally, false positive air bubbles may be observed in coronoid process fractures, which can be combined with a fractured zygomatic arch and zygomaticomaxillary complex.

3. True negative (TN)—no body/angle fracture + no AS in soft tissues.

**Figure 4:**
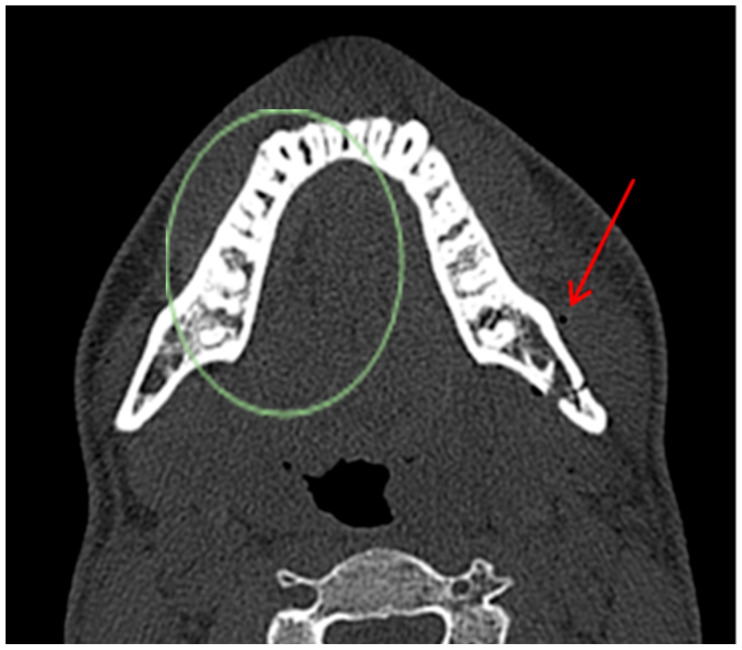
CT scan, horizontal view. A 22-year-old male patient diagnosed with a fracture of the left angle of the mandible with tooth 38 in the fracture line. The mechanism of injury was a hit to the chin, which could cause compound mandibular fractures. In this case, there was no combined fracture of the left angle + right body of the mandible. No fracture and AS were observed in the area of the body of the mandible on the right side, thus resulting in a true negative outcome (green circle). In this patient, a true positive result is also observed on the left side (red arrow).

In the case of a secondary fracture resulting from transmitted force directed at the mandibular body, it is reasonable to suspect a bone fracture within the mandibular body and air accumulation near this location. In the absence of air bubbles within the soft tissues surrounding the mandibular body or angle in indirect mechanism of injury and the absence of concomitant fractures at these locations, the true negative results were classified in terms of the given criteria (Figure 4). In addition, the majority of cases involving fractures of the condyle of the mandible are caused by an indirect mechanism. As a result, patients should undergo a comprehensive examination of the mandibular body and angle to prevent misdiagnosis and identify any additional fractures in this region.

4. False negative (FN)—diagnosed body/angle fracture + no AS in soft tissues.

**Figure 5:**
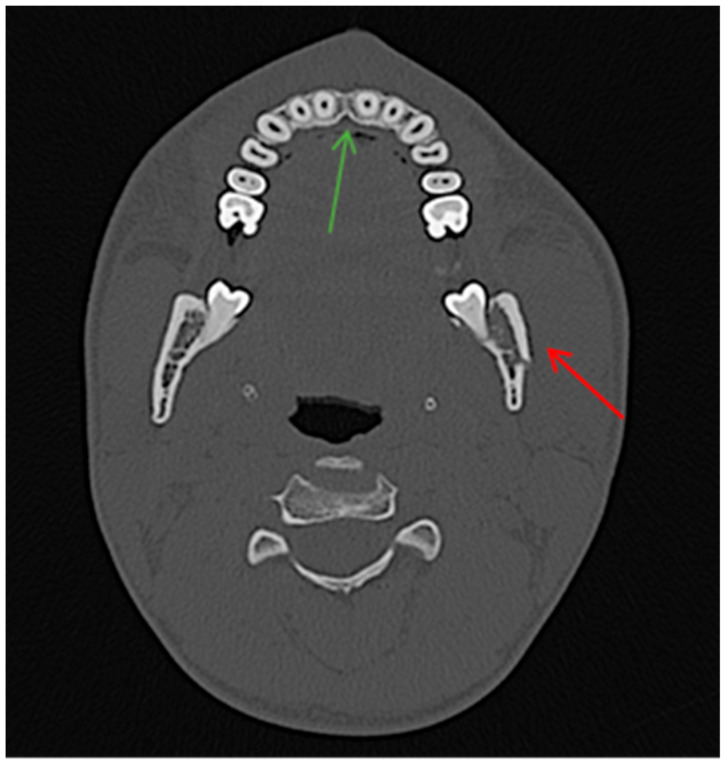
A 19-year-old male patient diagnosed with a displaced fracture of the left angle of the mandible with an impacted tooth in the fracture gap (red arrow). CT was performed 2 days after the injury. No air was observed in the region of the fracture. In the region of the anterior teeth, from the lingual side, the presence of air bubbles associated with saliva is observed (green arrow).

The delayed radiological examination postinjury resulted in the potential for air bubbles within the soft tissues to migrate from the fracture site or be absorbed rapidly as part of the physiological process. Consequently, these cases were classified as false negatives (Figure 5).

In accordance with the specified criteria, the following parameters were calculated:

- Sensitivity = TP/(TP+FN);
- Specificity = TN/(TN+FP);
- Positive predictive value (PPV) = TP/(TP+FP);
- Negative predictive value (NPV) = TN/(TN+FN);
- False positive rate (FPR) = FP/(FP+TN);
- False negative rate (FNR) = FN/(FN+TP).

## 3. Results

The study group consisted of 83.7% male and 16.3% female patients. Six patients were diagnosed with panfacial fractures. In the majority of cases, open reduction and internal fixation (ORIF) of mandibular fractures was required, performed under general anesthesia (81.4%). The characteristics of the analyzed patients are presented in Table 1. 

A thorough retrospective analysis of the preoperative diagnostic imaging (CT/CBCT scans) of 43 patients who underwent facial trauma affecting the mandible bone revealed a total of 71 fractures in different mandibular sites and air accumulation in the soft tissues, in proximity to the diagnosed fracture of the mandible. Open fractures were observed in 56%, while the remaining fractures were classified as closed. The number of TP results was identical in both established groups (*n* = 24). Similarly, with regard to the FP results, there was one case in each group. The TN results were primarily associated with open fractures under the previously described conditions. Conversely, FNs were more frequently observed in closed fractures (*n* = 3) than in open fractures (*n* = 1). The sensitivity, specificity, PPV, NPV, FPR, and FNR of the AS for the general study population and each fracture type are presented in Table 2.

## 4. Discussion

The proper distribution of stresses on the mandible is ensured by its spatial arrangement of force lines, which are referred to as Sicher’s trajectories. These are the lines of reinforcement that provide the greatest adaptation to the act of mastication. In addition, it is possible to identify areas that are most susceptible to injury and those that are most resistant. Bone resistance is reduced in three main areas: the area of the canine, the angle of the mandible, and the area of the neck of the condylar process. Champy’s research focused on the internal stresses that occur in the mandible during the process of mastication [10,11]. The studies demonstrate that the greatest compressive forces are observed at the inferior parts of the mandible, in proximity to the molars. The greatest degree of stretching occurs in the alveolar region of the mandible. Furthermore, the region surrounding the mandibular symphysis is distinguished by a twisting force that results in the separation of its constituent parts in a scissor-like manner [12,13].

The site of mandibular fractures depends not only on the reinforcements present in its structure, but also on the mechanism of injury. It should be noted that the frequency of fractures may vary depending on the center and country where the study was conducted. For example, in Washington (USA), where personal violence was the most common cause of injury, the most frequent fractures were in the angle (36%), body (21%), and mandibular symphysis areas (17%) [14,15]. On the other hand, in Turin (Italy), where falls and motorcycle accidents dominated, the most common fractures were the condyle (35%), the mandibular symphysis (26%), and the angle (25%) [16]. However, in Amsterdam (the Netherlands), where bicycle accidents are common cause of injury, the incidence of condylar fractures was 43%, followed by 25% for the mandibular symphysis and 16% for the mandibular body [16]. The presence and position of the wisdom teeth are also significant factors. In cases of trauma, the risk of mandibular angle fracture is increased by twice as much if the impacted wisdom teeth are present [17]. A 10-year study conducted in Washington demonstrated that up to 52% of patients presenting with a mandibular fracture had multiple fracture lines. The most common combination was a mandibular body and angle fracture (43%), followed by a mandibular body/symphysis with a mandibular condyle fracture (24%) [15]. The treatment of mandibular fractures in children and the elderly differs from that of adults. The primary challenge in examining these patient groups is the lack of effective communication and the unclear circumstances of the injury. In the case of pediatric patients, an additional obstacle is the necessity for CT, which often requires sedation. In children, the mandible is the most commonly fractured bone in the facial skeleton (36.9%), including the mandibular symphysis (27.9%). The location of the fracture is also related to the age of the pediatric patient. In younger patients, the mandibular condyle is more commonly fractured; whereas, in adolescents, the mandibular angle is the most frequently affected site [18]. In the evaluation of a child following an injury, a proper diagnosis is of significant importance. A study by Kannari et al. found that 14.8% of mandibular fractures in children are misdiagnosed, particularly in those under 13 years of age [19]. This may result in complications such as ankylosis, delayed development of the facial bones and teeth, with malocclusion [20]. In elderly patients (above 60 years old), approximately 20% of mandibular fractures are overlooked, particularly if the patient was in a hospital or nursing home at the time of injury [21]. These findings highlight the necessity for the development of additional diagnostic techniques to facilitate the accurate diagnosis of mandibular fractures. 

There are some studies that present clinical decision aids with recommendations for the exclusion of mandibular fractures. Especially the tongue blade bite test (TBT) with sensitivity of 98.5% and NPV of 98.7% serves as a valuable tool, which, when combined with clinical symptoms, might be comparably effective for radiological imaging [22,23]. It is believed that the validation of the test might be helpful in reducing unnecessary radiation exposure in some patient groups, maximizing the cost and time effectiveness. New diagnostic tools due to increasingly modern advancements in imaging technologies are being provided to maxillofacial trauma surgeons. While two-dimensional radiological techniques remain the primary method for identifying mandibular fractures in low-income countries, their utility is limited to isolated, simple fractures and often results in incomplete or incorrect diagnoses. Usually, several X-ray pictures need to be obtained at different projections to properly identify all visible fracture lines, as individual views are insufficient in detail, and some mandibular regions are poorly demonstrated in projections [4,5,24]. Therefore, CT scanning should be the preferred diagnostic tool over orthopantomography (OPG) for a more precise identification of the fracture lines. This method is preferred in all unstable patients when the mandibular fracture is suspected, or there is a clinically likely fracture not visualized on X-ray images [5,25]. When a sitting or standing position is impossible to achieve for a patient, which is common in severe polytrauma cases, the OPG and CBCT images cannot be obtained; yet, the CT is the best modality. Moreover, manipulations of two-dimensional CT scan data enable us to obtain the multiplanar (horizontal, sagittal, and coronal views) and 3D reconstructions. Thus, the issue of superimposed images of distant anatomical structures, presented in conventional plain radiography, can be reduced. In addition, 3D reconstructed images give surgeons a more realistic view of how the fracture might be treated during surgery, but some nondisplaced fractures may not be visible on 3D reconstruction. Dealing with multiple or comminuted facial fractures, CT/CBCT scans provide a helpful tool in identifying the precise location and direction of the fracture line, the extent and direction of displacement, and the degree of depression and rotation of bony fragments [5,9,25].

The identification of unrecognized mandibular fractures, which pose a significant risk to the patient, is an important part of the examination. In the event of an undiagnosed fracture, the affected area may become infected. Some surgeons postulate that any fracture that has persisted for a period exceeding 48 h is infected [26]. The direct presence of teeth and periodontal ligaments provides an environment conducive to bacterial growth in the fracture area. For this reason, open fractures are most often infected, particularly when a tooth is present in the fracture line. Moreover, the mobility of bone fragments and bone necrosis have been identified as contributing factors to increased inflammation [27]. In addition, patients with systemic diseases and patients with high scores on the mandibular injury severity score (MISS) are at an elevated risk of postoperative inflammatory complications following the treatment of mandibular fractures [28]. Ankylosis of the temporomandibular joint may be another consequence of an undiagnosed mandibular condyle fracture. Such complications may result in difficulties with speech and eating. The limited scope for effective oral hygiene in these patients provides an environment conducive to the development of periodontal disease. Furthermore, the growth of the facial bones is also disrupted, which results in significant asymmetry and malocclusion [29]. Long-term complications are defined as those occurring at least six weeks after the initial treatment. The most common risks include malocclusion, limited mouth opening, nerve dysfunction, pain, and facial asymmetry.

**Figure 6:**
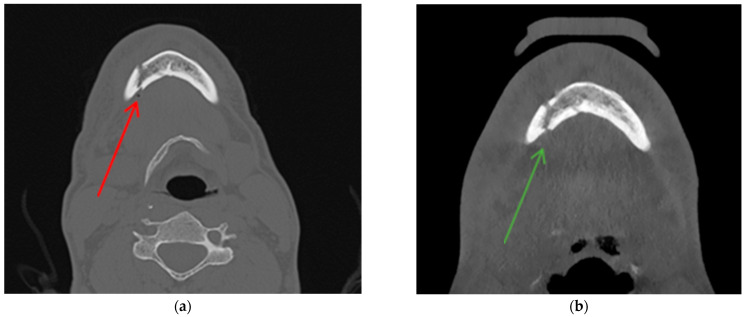
A 37-year-old patient presented with a fracture of the mandibular body on the right side and the left condylar process of the mandible. A CT scan was performed on the second day post injury (**a**). A CBCT scan was performed on the third day following the injury (**b**). The air bubble visible on the CT scan taken on the second day (red arrow) was already reabsorbed on the third day (green arrow). Therefore, we decided to reduce the number of patients and evaluate only the images taken on the second day after the injury.

CT and CBCT imaging are an effective method for detecting air bubbles in the soft tissues of the head and neck region, which can indirectly indicate the presence of fractures. In cases where AS appears in the body and angle of the mandible area, there is a higher possibility that a fracture may be present, despite being undetectable on CT/CBCT scans. This information can assist the surgeon in identifying potential fractures during the surgical procedure. However, further testing was necessary to substantiate this hypothesis clinically. In accordance with the findings of the preliminary study, the initial cohort of patients who met the eligibility criteria were individuals with CT/CBCT images taken within three days after the injury [9]. However, a detailed analysis of all patients’ radiographs revealed that, as early as the third day after the injury, the air bubble had either been reabsorbed into the soft tissues or had undergone a significant movement. In patients for whom it was possible to interpret more than one radiological examination taken at different time points prior to the ORIF procedure, a comparable pattern of air bubble change was observed (Figure 6). 

Based on these results, it can be concluded that CT/CBCT scans performed from the third day after the injury are not reliable. Accordingly, the study group was restricted to patients who had undergone a CT/CBCT scan within two days of the injury. The most significant challenge encountered during the course of the study was the unavailability of radiological documentation taken immediately following the injury. In CT/CBCT scans performed after an extended period, the AS was no longer present. However, time is not the sole limiting factor. It is crucial that the medical professional or radiologist analyzing the scans be aware of the air bubble’s positioning in relation to the tooth crowns to avoid any misinterpretation of the air present in the saliva. Furthermore, the presence of air in soft tissues in pneumothorax, zygomaticomaxillary complex, and Le Fort fractures requires careful consideration to ensure the accurate differentiation of AS from other regions in polytrauma patients. 

A number of scientific studies have demonstrated a direct correlation between the presence of air bubbles in bone injuries and the uninterrupted continuity of surrounding tissues. For example, a retrospective study by Fairbairn et al. reviewing CT scans of 16 hip dislocations in 15 patients revealed intracapsular air bubbles in 81% of patients who underwent CT within four hours of admission [30]. A significant limitation of AS is the time that has elapsed since the CT/CBCT scan was performed. In this instance, the presence of gas bubbles was discernible in only 25% of cases at the 48 h mark postinjury. It can, thus, be concluded that in the absence of penetrating trauma, the presence of intracapsular gas bubbles on CT scans is a reliable indicator of a recent hip dislocation [30]. Also, in closed pelvic fractures, a phenomenon analogous to AS has been observed, which has been defined as the vacuum phenomenon (VP). The occurrence of this phenomenon is contingent upon a number of factors, including the examination technique conducted, the joint position observed, and the characteristics of the studied population. It is noteworthy that the occurrence of VP in closed pelvic fractures is statistically significant [31]. Additionally, VP has been observed in numerous other joint regions, including the spine, sacroiliac joint, shoulder joint, knee joint, and temporomandibular joint [32,33]. This phenomenon is typically mild but may result in back and joint pain, particularly in the elderly [32,34]. In patients with a skull base injury, the AS sign has been demonstrated to be statistically significant for temporal bone fracture (*p* < 0.001). In this instance, the AS is situated within the temporomandibular joint region. In some cases, it may be the only symptom of a temporal bone fracture [35]. The presence of AS may prove beneficial not only in clinical contexts but also in the autopsy process. Following the performance of a postmortem CT (PMCT), it was demonstrated that the AS in relation to laryngeal fractures exhibited a sensitivity of 79.2%, a PPV of 95%, a specificity of 90.9%, a NPV of 34.5%, and an accuracy of 83% [36]. 

The sensitivity of OPG in detecting mandibular fractures is reported at 92%, which, in comparison to other X-ray projections with 66%, seems to have a more relevant clinical use in mandibular trauma patients than conventional radiographs [24]. Nevertheless, the limited utility of OPG caused by details obscurity is noted in detecting condylar fractures, especially with medial displacement. In comparison, CT techniques prove to identify mandibular lesions at up to 100% sensitivity, which is why it remains a favorable modality in many trauma departments [4,5]. Nevertheless, CBCT images show subtle changes in bone density and often mark fracture lines, making it difficult to identify fractures compared to CT. The evaluation of deep learning algorithms based on AI enables us to reach a precision of 97.8% and a sensitivity of 95.6% in detecting mandibular fractures based on CBCT scans [37]. In terms of open fractures of the mandible, the AS produced a sensitivity of 96% and NPV of 93.3%, which, in comparison to closed fractures cases (with a sensitivity of 88.9% and NPV of 57.1%, respectively), proved to be more useful in identifying the patients who did not have a fracture. While the PPV reached 96% in both types of fractures, specificity of the AS was noted to be higher in open fractures (93.3%) than in closed fractures of the mandible (80%), which means that the sign is more precise in detecting open mandibular fractures. The FNR was also higher in closed fractures (11.1%) than in open fractures (4%), which suggests that the AS is less useful in identifying the present closed fractures of the mandible than the open fractures. In 20% of closed fractures cases and 6.6% of open fracture cases, the results were incorrectly classified as positive, which means that the accuracy of the AS was lower regarding closed fractures. 

It is important to note that the presence of AS in the soft tissues surrounding the fracture area is an indirect symptom that is observed concurrently with the patient’s clinical examination. If AS is identified in the CT/CBCT scans prior to the ORIF procedure, it is advised that the attending physician conduct a comprehensive examination of the region corresponding to the air bubble during the surgical procedure whether there is a radiological fracture mark or not. To avoid unnecessary further invasive procedures, resulting from omitted fracture lines on the radiological images, it is essential to correctly diagnose additional fractures using alternative indirect indicators. If the bone fragments demonstrate increased mobility, direct surgical intervention will reduce the patient’s treatment costs, minimize any potential complications, and avoid questions being raised about the competency of the surgeon/radiologist interpretation of the CT/CBCT scans. Especially when these imaging techniques are standard procedures after head injuries, it seems crucial to have various tools for a proper and thorough interpretation.

What proves to be beneficial nowadays is AI with deep learning models or radiomics, which aim to reduce the prediction errors in radiological evaluation in a wide range of medical fields. The automated detection, segmentation, and classification of intricate patterns in both 2D and 3D images can be enhanced by AI tools [6,37]. Guermazi et al. revealed in his study that AI improved sensitivity from 64.8% to 75.2% in diagnosing fractures based on classic radiographs, and specificity improved from 90.6% to 95.6% in bone fractures in all anatomic regions except for the shoulder and/or clavicle and spine. Also, AI-assisted readings were on average 6.3 s faster than nonassisted ones [38]. As within the study not only musculoskeletal radiologists were included but also unspecialized medical trainees and specialists from other hospital departments analyzing radiographs in daily practice, it allowed for the generalization of the results. Regarding the mandibular fractures, for enhancing the effectiveness of CBCT image interpretation, some deep learning models, such as JawFracNet, have been created to improve both precision and sensitivity (97.8% and 95.6%, respectively), as well as reducing readout time by up to 2–3 times [37]. In the setting of busy emergency departments, readouts are frequently required during night shifts, which were proved to recede in quality after 4 p.m. with the highest rate of missed fractures at 3 a.m. [38].

In that case, new indirect indicators incorporated within radiological interpretation would give a more precise analysis and diagnosis, higher fluency of clinicians in interpreting the images, and reduced number of underdiagnosed fractures resulting from human imperfection. The proposed “air sign” in this study would provide additional help to the AI models so as to diagnose mandibular fractures more accurately.

## 5. Study Limitations

This analysis aims to enhance diagnostic performance in mandibular fractures cases; however, it has its limitations. As this phenomenon has not been described concerning mandibular fractures in any previous works so far, there was no relevant evidence to comparatively support our finding. Hence, the radiological significance, clinical utility, and possible interoperation with existing diagnostic capabilities were described. The other limitation was the small size of the study group. Not all patients with mandibular fractures had the CT/CBCT of the head performed within 2 days postinjury; therefore, it was not possible to consider these cases, which resulted in a reduced number of patients included. The next variable that may influence the overall results is the human-performed collection of medical data and the interpretation of radiological images. Errors in radiological interpretation by humans may arise from missing a fracture line or air accumulation due to insufficient individual experience or demanding schedules that many clinicians face in different hospital facilities. As a result, there are still subsets of the fractures that remain overlooked with the use of CT/CBCT scanning. In that matter, new indirect indicators incorporated within radiological interpretation would give a more precise analysis and diagnosis, higher fluency of surgeons in interpreting the images, and reduced number of underdiagnosed fractures resulting from human imperfection. 

## 6. Conclusions

The clinical value of AS in mandibular fractures with clearly visible dislocation on CT/CBCT is limited. The AS has a practical use in finding mandibular fractures without dislocation. Direct signs of mandibular fractures and the presence of AS on the opposite side of the body/angle of the mandible may indicate an additional fracture without dislocation, which may be invisible on 3D reconstruction. The sensitivity and specificity of AS are lower than that of OPG, CT, and CBCT, but it is an important additional radiological indicator. When integrated with a comprehensive clinical examination of the patient, radiological image interpretation, and even the use of AI, it can effectively reduce the risk of misdiagnosing mandibular body and angle fractures without dislocation.

## Figures and Tables

**Table 1:** Characteristics of the study group.

Parameter	Number/Percent
***Age***	
Mean (±SD ¹)	35.37 (±16.94)
***Sex***	
Male	36 (83.7%)
Female	7 (16.3%)
***Location of fractures***	
Isolated fractures of the mandible	37 (86.1%)
Panfacial fractures	6 (13.9%)
***Treatment***	
*ORIF* ²	35 (81.4%)
Conservative (MMF ³)	8 (18.6%)
***Type of fracture***	
Open	40 (56%)
Closed	32 (44%)

¹ SD—standard deviation; ² ORIF—open reduction internal fixation; ³ MMF—mandibulomaxillary fixation.

**Table 2:** Statistical analysis of the air sign (AS) in open and closed fractures of the mandible.

Fracture Type	Number of Fractures	TP Results	TN Results	FP Results	FN Results	Sensitivity (%)	Specificity (%)	Positive Predictive Value (%)	Negative Predictive Value (%)	False Positive Rate (%)	False Negative Rate (%)
Total	71 *	47 *	18	2	4	92.2%	90.0%	95.9%	81.8%	10.0%	7.8%
Open fractures	40	24	14	1	1	96.0%	93.3%	96.0%	93.3%	6.6%	4.0%
Closed fractures	32	24	4	1	3	88.9%	80.0%	96.0%	57.1%	20.0%	11.1%

* One patient classified for both analyzed fracture groups because of simultaneous unilateral fracture of mandibular angle and ramus.

## Data Availability

The data presented in this study are available on request from the corresponding author. Data are not publicly available due to privacy.
